# A Systematic Review of Advanced Drug Delivery Systems: Engineering Strategies, Barrier Penetration, and Clinical Progress (2016–April 2025)

**DOI:** 10.3390/pharmaceutics18010011

**Published:** 2025-12-22

**Authors:** Assem B. Uzakova, Elmira M. Yergaliyeva, Azamat Yerlanuly, Zhazira S. Mukatayeva

**Affiliations:** 1 Department of Chemistry, Faculty of Natural Sciences and Geography, Abai Kazakh National Pedagogical University, Almaty 050010, Kazakhstan; azaraze8575@mail.ru (A.Y.); jazira-1974@mail.ru (Z.S.M.); 2 Department of Natural Science Disciplines, U. Sultangazin Pedagogical Institute, Akhmet Baitursynuly Kostanay Regional University, Kostanay 110000, Kazakhstan

**Keywords:** drug delivery systems, nanocarriers, liposomes, lipid nanoparticles, biological barriers, AI-assisted design

## Abstract

**Background/Objectives:** Advanced drug delivery systems (DDSs) are essential for targeted delivery, controlled release, and reduced systemic toxicity, but their clinical adoption is limited by biological barriers, manufacturing complexities, and cost. The aim of this systematic review is to critically evaluate the quantitative relationships between platform design, overcoming biological barriers, and clinical translation outcomes for DDS developed between 2016 and 2025. **Methods**: A comprehensive literature search was conducted in PubMed/MEDLINE, Scopus, and Web of Science (January 2016–April 2025) in accordance with the PRISMA 2020 guidelines. Included studies focused on experimental or clinical data for nanocarrier platforms (liposomes, lipid nanoparticles, polymer systems, biomimetic carriers, extracellular vesicles). Data on platform characteristics, interactions with barriers, pharmacokinetics, manufacturing, and clinical outcomes were extracted and synthesized in narrative form due to the significant methodological heterogeneity. **Results**: An analysis of 77 included studies confirms that successful clinical translation depends on matching the physicochemical properties of the carrier (size, surface chemistry, material) to specific biological barriers. Liposomes and lipid nanoparticles (LNPs) remain the most clinically validated platforms, exploiting the EPR effect and liver tropism, respectively. Key engineering solutions include stealth coatings, ligand-mediated targeting, and stimulus-responsive materials to overcome barriers such as mononuclear phagocyte system clearance, the blood–brain barrier, and mucosal barriers. Microfluidic and continuous manufacturing processes enable reproducibility, but scalability, cost, and immunogenicity (e.g., anti-PEG responses) remain key translational challenges. Engineered extracellular vesicles, biomimetic carriers, and 3D/4D-printed systems combined with AI-driven design demonstrate the potential for personalized, adaptive delivery. **Conclusions**: Cutting-edge DDSs have validated their clinical value, but realizing their full potential requires a holistic, patient-centered design approach integrating barrier-specific engineering, scalable manufacturing, and rigorous safety assessment from the earliest stages of development. Further progress will depend on standardizing methods for new platforms (e.g., extracellular vesicles), implementing digital and AI tools, and ensuring translational feasibility as a fundamental principle.

## 1. Introduction

Advanced drug delivery systems (DDSs) address the limitations of traditional administration routes, including poor bioavailability, degradation, and off-target toxicity [1]. They achieve this by enabling optimized pharmacokinetics and site-specific targeting through principles of precision nanoparticle design [2,3].

The tunable properties of nanocarriers such as liposomes, polymeric nanoparticles, LNPs, dendrimers, and micelles have facilitated their clinical translation, with numerous formulations approved since the first FDA-approved nanodrug, Doxil^®^, in 1995 [4].

Although the enhanced permeability and retention (EPR) effect promotes tumor accumulation [5], its heterogeneity limits reliability [6,7]. This has spurred advancements in both manufacturing (e.g., microfluidics [8]) and targeting strategies (e.g., ligand-mediated approaches [9].

Liposomes and LNPs represent the most clinically advanced platforms. Liposomes benefit from these manufacturing innovations [8] and surface modifications such as pegylation [9] for optimized chemotherapeutic delivery. LNPs enable mRNA vaccines [10,11] and siRNA therapies [12], while polymeric systems including PLGA-based nanoparticles provide controlled release and combination delivery [13].

Despite these advances, clinical translation continues to face persistent barriers, including mononuclear phagocyte system (MPS) clearance [2,3], manufacturing scalability issues [8], and regulatory complexity, requiring integrated computational and interdisciplinary approaches [2,3]. The diversity of modern nanoscale carriers demonstrates that their efficacy is determined by the precise interaction between nanostructure, surface chemistry, material composition and biological processes [2,3]. Accumulating clinical and translational data support a key principle: optimal therapeutic efficacy is achieved when platform characteristics are specifically designed to match specific biological barriers such as vascular permeability, immune surveillance, and cellular uptake mechanisms [9,10,14].

Liposomes are the most clinically proven nanocarriers, whose efficacy is determined by membrane composition [15], size (70–120 nm), and surface modifications such as PEGylation [9]. Modern microfluidic manufacturing enables reproducible control of these parameters, which, as demonstrated for PEGylated liposomal doxorubicin (Doxil^®^), leads to improved pharmacokinetics and safety profile [8,9].

LNPs are the current standard for nucleic acid (siRNA, mRNA) delivery. Their effectiveness is based on ionizable lipids, which ensure endosomal exit, and a natural tropism for hepatocytes via apoE-mediated uptake [10]. Accessory lipids (DSPC, cholesterol) provide structural integrity, while PEG-lipids regulate stability and circulation time [10,16]. Microfluidic mixing parameters directly determine encapsulation efficiency and particle size, making experimental design approaches critical for scalable GMP manufacturing [16]. Clinically validated examples such as patisiran (siRNA) [12] and the mRNA vaccine BNT162b2 [10,11] demonstrate how chemistry, process control, and platform engineering enable the creation of effective gene-modulating therapeutics. Preclinical development is also aimed at improving key characteristics such as thermal stability [17].

Biodegradable polymeric carriers (PLGA, PLA, PCL) provide controlled release and the possibility of surface functionalization. Release kinetics depend on polymer crystallinity, molecular weight, and erosion mechanisms [13,18]. Polymeric micelles effectively solubilize hydrophobic drugs and can incorporate stimulus-responsive components [19]. Key parameters for translation include residual solvent levels, micelle stability below the CMC, and confirmation of polymer identity. Functionalized systems, such as those with ascorbic acid, show potential for targeted brain delivery, and continuous manufacturing processes help address scalability issues [20].

Dendrimers provide precise multivalency and control of ligand density but require surface modification to reduce cytotoxicity [21]. Mesoporous silica nanoparticles (MSNs) are characterized by high loading capacity and tunable pores for controlled release [22], but their long-term biodegradation and clearance remain challenging [23].

Biomimetic carriers (e.g., cell membrane-coated) inherit biological markers to reduce immune recognition and homotypic targeting [24]. Extracellular vesicles (EVs, exosomes) exhibit natural tropism, low immunogenicity, and the ability to deliver proteins and nucleic acids [25,26]. Despite their potential, their widespread clinical adoption is hampered by difficulties in standardizing isolation, reproducible loading, and scaling up production.

This systematic review aims to critically synthesize and evaluate the current evidence (2016–2025) in the field of advanced DDS, with a focus on identifying quantitative relationships between platform design, overcoming biological barriers, and clinical translation outcomes. Unlike previous narrative reviews, this work presents a structured analysis conducted in accordance with the PRISMA guidelines that systematically correlates engineering solutions (stealth coatings, ligand-mediated targeting, and stimulus-responsive materials) with specific physiological barriers (e.g., BBB, tumor microenvironment, mucociliary clearance). By integrating preclinical mechanistic data with clinical trial results, we identify not only optimal technology platforms but also persistent translational bottlenecks in manufacturing, scalability, and safety assessment. This review also highlights the emerging paradigm shift toward intelligent adaptive systems, including biomimetic carriers, engineered extracellular vesicles, and AI-optimized formulations, and evaluates their potential to overcome the limitations of traditional nanocarriers. Ultimately, we offer a forward-looking perspective that positions translational feasibility and patient-centered design as essential foundational principles for the next generation of drug delivery platforms.

## 2. Materials and Methods

### 2.1. Literature Search Strategy and Data Sources

A comprehensive and systematic literature search was conducted in the electronic databases PubMed/MEDLINE, Scopus, and Web of Science for the period from January 2016 to April 2025 (search date: April 2025). Search strategies were developed using combinations of controlled vocabulary terms and free-text keywords related to drug delivery and nanomedicine, including but not limited to “drug delivery systems,” “nanoparticles,” “liposomes,” “lipid nanoparticles,” “polymeric nanoparticles,” “extracellular vesicles,” “biomimetic delivery,” “biological barriers,” and “clinical translation.”

The study protocol was developed a priori (see Supplementary File S1). This systematic review was prospectively registered on the Open Science Frame-work (OSF) on 17 December 2025, with the associated project available at https://osf.io/36qws (accessed on 17 December 2025). The results were reported in accordance with the PRISMA 2020 Guidelines (Preferred Reporting Items for Systematic Reviews and Meta-Analyses) to ensure transparency and completeness of data reporting [27]. Since the planned synthesis is qualitative (narrative) and does not include quantitative meta-analysis, the Synthesis Without Meta-Analysis (SWiM) Guidelines were also used to describe it [28].

Reference lists of included articles and relevant reviews were additionally screened to identify potentially eligible studies not captured by the initial search. The complete electronic search strategies, including all search strings used for each database, are provided in Supplementary File S2 (Full electronic search strategy).

### 2.2. Eligibility Criteria and Study Selection

Study eligibility was defined a priori according to predefined inclusion and exclusion criteria.

- The PICOS criteria were used to determine study inclusion:
- Study Design: Original experimental, preclinical (in vivo, ex vivo), or clinical studies.
- Population/Intervention: Studies focused on the development, characterization, preclinical, or clinical evaluation of advanced drug delivery systems (DDSs), such as lipid and polymer nanoparticles, exosomes, micelles, 3D-printed systems, and other innovative platforms.
- Comparison: This could include comparison with control groups (e.g., free drug), different DDS, or routes of administration, but was not a mandatory criterion.
- Outcomes: Key physicochemical characteristics of the DDS, efficacy data (in vitro*,* in vivo), pharmacokinetics, safety/toxicity, clinical outcomes, or explicitly described translational significance.
- Articles published in English.
- Exclusion criteria:
- Purely conceptual, opinion-based, or hypothesis articles without original experimental data;
- Studies lacking sufficient characterization of the delivery system or relevant outcome measures;
- Conference abstracts, editorials, and non–peer-reviewed sources.

**Figure 1:**
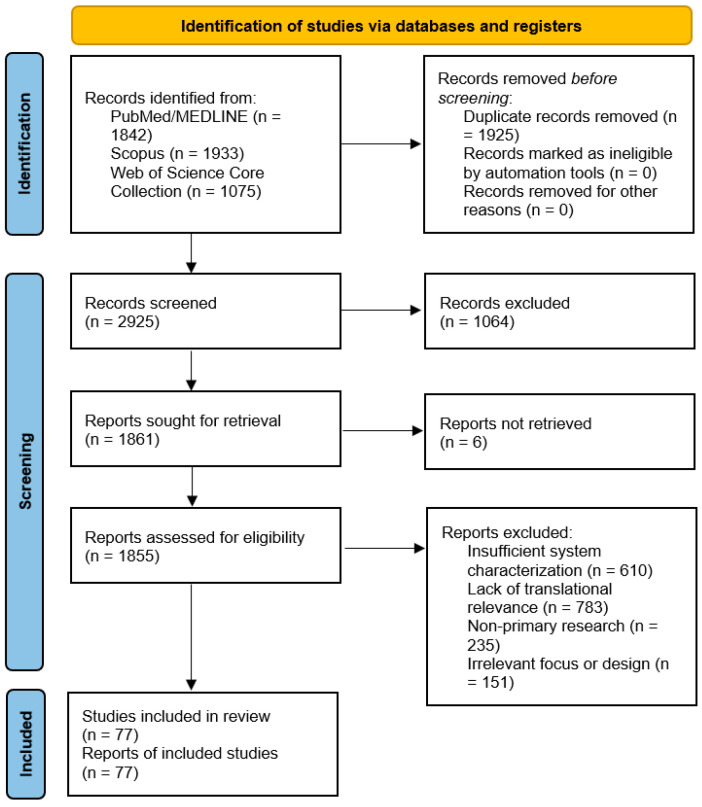
PRISMA 2020 flowchart depicting the study selection process for this review.

Study selection was performed in two stages: (i) screening of titles and abstracts, followed by (ii) full-text assessment for eligibility. The study selection process is summarized using a PRISMA 2020 flow diagram (Figure 1).

### 2.3. Data Extraction and Assessment of Study Quality and Translational Relevance

Data were systematically extracted from all included studies using a standardized extraction framework. Extracted information included: authors and year of publication; type of delivery platform; physicochemical and formulation characteristics; route of administration; therapeutic cargo; experimental model; key outcomes; and reported limitations.

Extracted data were organized into comprehensive summary tables (Table 1 and Table 2) and thematic matrices that formed the basis for the comparative narrative synthesis. Detailed table for all included studies are provided in the Supplementary File S3.

Methodological quality and potential sources of bias were assessed using an adapted qualitative framework informed by established risk-of-bias tools for preclinical and translational research (including SYRCLE principles, where applicable). The assessment focused on criteria most relevant to drug delivery research, including adequacy of carrier characterization, use of appropriate controls, relevance of experimental models, reproducibility of outcomes, and evaluation of toxicity and immunogenicity. Rather than excluding studies based on quality alone, these assessments were used to contextualize the strength and translational robustness of the evidence.

### 2.4. Data Synthesis and Organization of Results

Due to confirmed heterogeneity across delivery platforms, biological targets, routes of administration, and outcome measures, statistical pooling of results was not feasible. Therefore, a structured narrative synthesis was conducted.

Studies were synthesized and organized into predefined thematic domains, reflecting key determinants of drug delivery performance and clinical translation. These domains included: (i) delivery platform type; (ii) routes of administration and device–formulation strategies; (iii) biological barriers and engineering solutions; (iv) manufacturing, scale-up, and quality-by-design considerations; (v) safety, immunogenicity, and translational evaluation; and (vi) clinical performance and real-world implementation. This approach enabled systematic comparison of evidence while preserving the mechanistic and translational context of diverse DDS technologies.

In preparing this paper, generative AI tools (ChatGPT, GPT-5 model, OpenAI) were used solely as a language aid—to refine wording, perform stylistic editing, and improve clarity. Generative AI was not used to generate data, interpret it, or formulate scientific conclusions. All scientific decisions, analysis results, and final content of the article were independently completed and reviewed by the authors.

## 3. Results

### 3.1. Results of the Systematic Search and Study Selection

A systematic search of three electronic databases (PubMed/MEDLINE, Scopus, and Web of Science Core Collection) conducted up to 15 April 2025 initially yielded 4850 records (Figure 1). After removal of 1925 duplicates, 2925 unique records underwent title-abstract screening.

The first screening phase led to the exclusion of 1064 records, primarily for being non-original publication types (e.g., conference abstracts, editorials; n = 321) or falling outside the review’s scope (e.g., diagnostic applications without therapeutic intent; n = 461).

The remaining 1861 full-text articles were sought for retrieval. Of these, 6 reports could not be retrieved despite exhaustive efforts via institutional subscriptions and inter-library loan services. The remaining 1855 articles were rigorously assessed against our eligibility criteria. A significant majority (n = 1784) were excluded at this stage. The two most frequent reasons for exclusion were: (1) a lack of translational relevance (purely in vitro studies without disease models or scalability discussion; n = 783), and (2) insufficient physicochemical characterization of the drug delivery system (n = 610). Other common reasons included non-primary research formats and studies with an irrelevant focus (e.g., environmental nanotoxicity).

Ultimately, 77 studies satisfied all inclusion criteria and formed the basis for the qualitative synthesis in this review. The complete selection process is detailed in the PRISMA 2020 flow diagram (Figure 1).

### 3.2. Synthesis by Engineering Strategies to Overcome Barriers to Different Routes of Administration

A systems analysis of the included studies demonstrates that the clinical success of a delivery system is determined by its ability to sequentially overcome a cascade of interconnected barriers—from initial barriers specific to the chosen route of administration to fundamental biological limitations common to most nanocarriers. This section presents a thematic synthesis of the data, organized by major routes of administration and key biological targets. For each route, we analyze: (1) specific physicochemical and anatomical barriers; (2) engineering solutions at the device, formulation, and material properties level designed to overcome them; and (3) interactions with subsequent intrinsic and systemic barriers in the body. This integrated approach allows us to trace the entire pathway of a therapeutic agent “from the point of administration to the point of action,” identifying critical points where engineering design must be aligned with the biology of the target tissue.

#### 3.2.1. Transdermal Delivery: From Passive Diffusion to Active Microdevices

Barrier: Limited permeability of the stratum corneum for most therapeutic agents.

Engineering strategy (Device): Microneedles (MNs), which create temporary microchannels, represent the most clinically advanced active transdermal delivery platform [30,36]. Key design parameters include geometry (length, shape) to control penetration depth and material (e.g., soluble polymers), which determine mechanical strength and release kinetics. Complex architectures, such as bilayer systems, allow for separation of the administration and sustained release stages [37].

Interaction with internal barriers: Delivery efficiency is determined not only by the microchannel creation but also by the subsequent fate of the drug in the skin tissue. For example, in vaccination, studies show that soluble microneedles can effectively deliver antigens, inducing both humoral and cellular immune responses comparable to intramuscular injection, confirming their ability to overcome local tissue barriers and interact with the immune system [30,36]. Controlling the release (e.g., using PLGA particles encapsulated in MNs) allows for optimization of the drug exposure profile [36].

Interaction with systemic barriers: After release in the dermis, nanocarriers intended for systemic action drain into lymphatic and blood capillaries. Here, their fate is determined by the dynamic formation of a protein corona, the composition of which depends on the surface properties of the carrier and ultimately regulates its recognition by the immune system and circulation time [38,39].

#### 3.2.2. Pulmonary Delivery: Co-Engineering Aerodynamics and Surface Engineering to Overcome Mucociliary Clearance

Barrier: Effective pulmonary delivery is hampered by a cascade of physical and biological barriers: (1) aerodynamic sedimentation, which requires an optimal particle size to reach the alveoli; (2) mucociliary clearance, which rapidly removes particles adhered to mucus; (3) phagocytosis by alveolar macrophages.

Engineering strategy (Device + Shape):

Aerodynamic Optimization and Devices: For deep lung deposition, an aerodynamic particle diameter in the range of 1–5 μm is critical [40]. Current strategies include the development of dry powder inhalers (DPIs), where particles are purposefully created as highly dispersed nanoagglomerates. Such systems, optimized using design of experiments (DoE), exhibit excellent aerodynamic properties (e.g., MMAD~1.7 μm, FPF > 70%) and enable efficient delivery of drugs such as paclitaxel to lung cells [41].

Surface engineering for mucus clearance: The key to increasing residence time is the creation of mucoinert surfaces that minimize interaction with mucin. A comparative experimental study of various protein nanoparticle surface modifications showed that polyethylene glycol (PEG) coating and layer-by-layer (LbL) assembly of a zwitterionic layer provide the fastest transport in nasal mucus, while cationic particles exhibit strong mucoadhesion [40,42]. These data are consistent with previous findings demonstrating that dense PEGylation allows nanoparticles to distribute uniformly in the airways and avoid rapid clearance [40].

Interaction with internal barriers:

After overcoming the mucus barrier, nanocarriers encounter cellular barriers. Surface engineering directly influences this step. Thus, cationic and LbL-modified particles, despite inferior mucus penetration, demonstrate increased uptake by epithelial and dendritic cells in vitro in the presence of mucus, which may be advantageous for vaccines [42]. However, for most therapeutic strategies requiring prolonged retention or penetration into the epithelium, mucoinertness (achieved by PEG or zwitterionic coatings) remains a priority as a way to delay clearance by macrophages and ensure access to target cells [40,42]. Thus, the choice of surface modification strategy represents a compromise between the rate of transport in mucus and the efficiency of subsequent cellular uptake.

#### 3.2.3. Intranasal Delivery to the CNS: Navigating Anatomy and Biology

Barrier: The effectiveness of the intranasal route for CNS delivery is limited by rapid mucociliary clearance and the need for drug deposition in the olfactory region to access direct neuronal pathways (olfactory and trigeminal nerves). Quantitative studies confirm that this route is dose-dependent and effective for delivering even large biological molecules, such as antibodies [43].

Engineering strategy (Device + Form):

Retention (Overcoming Clearance): Bioadhesive and mucoretentive systems are used to increase mucosal contact time and counteract clearance. Bilosomes incorporated into an in situ gel demonstrated an 11.7-fold increase in the bioavailability of zolmitriptan in the brain compared to intravenous administration and ensured nearly complete (~98%) direct nose-to-brain transport [31]. Similarly, thermoreversible mucoadhesive gels significantly increased the bioavailability and direct transport rate of the antiepileptic drug levetiracetam [44].

Transport and transcytosis: To enhance specific uptake and penetration along neuronal pathways, nanocarriers are functionalized with ligands. PLGA nanoparticles modified with the RVG29 peptide demonstrated enhanced delivery to the trigeminal nerve and adjacent CNS regions, providing spatial targeting [45]. Modified chitosan micelles with ligands (mannose, Tat) efficiently delivered plasmid DNA to the brain, demonstrating higher gene expression compared to the intravenous route [46]. Polymeric nanoparticles conjugated with a ligand increased paclitaxel concentration in the brain by 5.6-fold after intranasal administration [47].

Carrier Integrity: Mechanistic studies using FRET technology confirmed that nanoemulsion cores in chitosan-coated nanocapsules are able to cross the nasal epithelial barrier intact, highlighting the role of chitosan in facilitating transport without degradation of the carrier [48].

Interaction with Intrinsic Barriers:

Although this strategy effectively bypasses the blood–brain barrier (BBB), it requires careful safety assessment. Direct visual tracking methods (e.g., using gold nanorods) confirmed rapid transport of nanomaterials from the nose to the brain, but also revealed their accumulation in the olfactory bulb [49]. This highlights the need to optimize delivery systems to minimize potential long-term neurotoxicity and neuroinflammation associated with particle accumulation.

#### 3.2.4. Oral and Mucosal Delivery: Engineering to Overcome Chemical, Mucosal, and Epithelial Barriers

Barrier: Oral delivery faces the most challenging cascade of barriers: (1) chemical degradation in the acidic environment of the stomach and by proteolytic enzymes; (2) a dense and renewing mucus layer that limits access to the epithelium; (3) a network of tight junctions between enterocytes that impedes paracellular transport of macromolecules.

Engineering strategy (Formulation + Material Properties):

Enhancing the solubility and absorption of lipophilic substances: For small molecules with low aqueous solubility, the use of lipid nanosystems, such as self-nanoemulsifying drug delivery systems (SNEDDSs), is an effective strategy. They significantly increase oral bioavailability and prolong the half-life of drugs, as demonstrated for chlorpromazine [50].

Penetrating the mucus barrier: A key challenge is minimizing particle adhesion to mucin. Studies show that creating muco-inert surfaces using a high-density polyethylene glycol (PEG) coating ensures penetration of nanoparticles through mucus regardless of pH [51]. Comparative analysis of various surface modifications of protein nanoparticles confirms that PEG coating and layer-by-layer (LbL) zwitterionic shells provide the fastest transport in mucus, while cationic surfaces lead to strong mucoadhesion [42]. This principle is universal for all mucous membranes.

Enhanced transepithelial transport for biologics: Delivery of peptides, proteins, and antibodies requires strategies that transiently increase epithelial permeability. Anionic silica nanoparticles reversibly modulate tight junctions through binding to integrins, providing significant bioactivity for insulin and exenatide after oral administration [52]. Combined systems, such as PEG-coated albumin nanoparticles with a safe permeation enhancer, achieved breakthrough (~3.7%) oral bioavailability of the monoclonal antibody bevacizumab [32].

The role of carrier mechanical properties: In addition to surface chemistry, particle elasticity is critical. Nanoparticles with tuned elasticity combined with a biomimetic coating demonstrate significantly higher transcytosis efficiency across intestinal epithelium compared to rigid counterparts [53].

Interaction with internal and systemic barriers:

Successful delivery through mucosal surfaces depends on the system’s ability to act locally and reversibly, without causing permanent damage or systemic toxicity. However, after crossing the epithelium, the nanocarrier enters the systemic circulation, where its fate is determined by a fundamental barrier—the dynamic formation of a protein corona. Studies on soft polymer nanomaterials show that it is the constant remodeling, rather than the initial composition of the corona, that controls their circulation time in the blood [38]. Moreover, the “stealth effect” of PEG depends on the adsorption of specific proteins that form a protective biomolecular corona [39]. Importantly, the composition of plasma, and therefore the corona, changes in diseases (cancer, inflammation), which can directly affect delivery efficiency and should be considered when designing personalized therapies [54]. Thus, engineering for mucosal pathways must be two-tiered: the first tier addresses local issues of the gastrointestinal tract or vaginal tract, while the second tier prepares the host for interaction with the universal circulatory and immune surveillance system.

#### 3.2.5. Ocular Delivery: Improving Ocular Retention and Penetration

Barrier: Topical, periocular, and intravitreal delivery are limited by rapid tear turnover, dense corneal epithelium, and slow vitreous diffusion, resulting in extremely low bioavailability.

Engineering strategy (Device + Formulation):

Nanoscale carriers and in situ gels: LNPs, polymeric micelles, and dendrimers have demonstrated improved precorneal retention and corneal penetration while maintaining optical clarity and patient comfort [55]. In situ gelation systems provide sustained release on the ocular surface.

Long-acting intravitreal implants: Sustained-release systems have been developed for the treatment of chronic posterior pole diseases (e.g., age-related macular degeneration, uveitis). A landmark example is the ranibizumab Port Delivery System (PDS), the first FDA-approved refillable intravitreal implant system, which provides continuous drug delivery and allows for extended dosing intervals of up to 6 months in pivotal clinical trials [29].

Device-formulation design: Sophisticated platforms are being developed for delicate intravitreal delivery, such as injectable, photosensitive implants loaded with nanoparticles that provide controlled release [56], and hybrid lipid-polymer platforms [55].

Interaction with internal barriers:

The main concerns after administration are biocompatibility and minimizing the inflammatory response in the sensitive tissues of the eye. Long-lasting implants and nanoparticles must be designed to avoid toxicity to photoreceptors and retinal cells. Furthermore, for nanocarriers that can drain into the systemic circulation via the uveoscleral route or after intravitreal administration, the general principles of interaction with the protein corona and the immune system, described in the previous sections, apply. Thus, ophthalmic systems require a special balance between duration of action, local safety, and formulation stability.

#### 3.2.6. Tumor and Brain Targeting: Strategies Against Selective Biological Barriers

Barrier: Delivery to solid tumors and across the BBB represents the highest level of challenge due to the selectivity of these biological interfaces. The tumor stroma creates physical (dense extracellular matrix) and physiological (increased interstitial pressure) resistance, while the BBB tightly controls molecular trafficking into the CNS through endothelial tight junctions and the expression of efflux pumps.

Engineering strategy (Targeting + Overcoming):

Mechanisms of tumor penetration: Basic research using various nanoparticles has shown that their penetration into tumors occurs predominantly through interendothelial clefts rather than by transcytosis, clarifying our understanding of the EPR effect [57].

Active tumor microenvironment (TME) remodeling: A rational approach is to combine cytotoxic nanocarriers with TME-modifying agents. For example, co-administration of doxorubicin nanoparticles with a TGF-β inhibitor resulted in normalization of the stroma of triple-negative breast cancer, decreased fibrosis, and enhanced antitumor immunity, improving overall delivery [58].

Engineered carriers for barrier disruption: To directly penetrate the dense extracellular matrix, nanoparticles carrying enzymes (e.g., collagenase) have been developed that locally degrade collagen, significantly enhancing the penetration of subsequent chemotherapeutic nanoparticles [59].

Multistage (“smart”) systems: Systems with detachable components are being developed to overcome sequential barriers. For example, nanoparticles that initially target tumor vessels and deplete the extracellular matrix, and then, upon detaching, directly attack cancer cells [60].

Targeting the BBB and its limitations: The main strategy for delivery to the CNS is the functionalization of carriers with ligands to receptors on the BBB endothelium (e.g., transferrin). However, a key study [61] demonstrated that in vivo formation of a protein corona on the surface of such targeted particles masks the ligand and almost completely inhibits receptor-mediated transcytosis, dramatically limiting the effectiveness of this approach.

Interaction with internal barriers:

Even for the most sophisticated engineering solutions, the protein corona remains a crucial and often ignored barrier that can nullify the intended targeting function [61]. This highlights the need to move toward “corona-aware” design. Furthermore, the high heterogeneity of both tumors and BBB status among patients requires personalized approaches. Machine learning methods are beginning to be applied to predict the effectiveness of nanoparticle delivery to tumors based on their physicochemical properties [62], paving the way for rational in silico screening. Accounting for disease-related plasma variability is also becoming a critical factor for in vivo predictability [54].

#### 3.2.7. Conclusion: Integrated Design and Future Directions

A systematic synthesis of evidence (2016–2025) identifies a central tenet of modern DDS design: clinical success is determined not by the maximum complexity of a single component, but by the optimal integration of engineering solutions that predictably overcome the full cascade of barriers “from device to target.”

1. From route of administration to systemic fate: Efficacy begins with co-engineering the device and formulation to overcome the anatomical and physiological limitations of a specific route (transdermal microneedles [30,36,37], aerodynamic powders [41], nasal sprays and gels [31,44]. However, once the carrier overcomes the first epithelial barrier, its fate is dictated by a universal interface in vivo—the dynamically forming protein corona [38,39]. Ignoring this step undermines even the most sophisticated targeting strategies [61].
2. Evolution of targeting paradigms: In the field of tumor delivery, the focus has shifted from passive accumulation (EPR) to active remodeling of the microenvironment [34,58] and the creation of multistage systems [60]. For the BBB, it is becoming clear that simple ligand binding is not enough—strategies for managing or evading the protein corona are required.
3. Key challenges and tools: Personalization (taking into account the disease state affecting the corona [54], scalability (see Section 3.3), and predictability are coming to the forefront. The integration of machine learning and in silico models for predicting in vivo behavior based on physicochemical properties opens the way to accelerated and rational design [62].
4. The future: The next generation of platforms will not just be drug carriers, but adaptive therapeutic systems. They will combine precision material properties (elasticity [53], shape [63], “corona-aware” surface design, means of overcoming tissue barriers, and, possibly, logic for responding to biological signals. Their development will require an unprecedented convergence of pharmaceutical engineering, computational biology, and clinical science to translate laboratory breakthroughs into accessible and effective therapies for patients.

### 3.3. Synthesis of Manufacturing, Scale-Up, and Quality-by-Design Considerations

An analysis of the included studies on delivery system development reveals a distinct evolution: while early studies focused predominantly on proof of concept and biological efficacy, modern publications necessarily address manufacturing and scale-up issues as an integral part of platform design. This paradigm shift reflects a growing recognition that reproducibility and cost-effectiveness of manufacturing are determinants of clinical success. Below, we present a synthesis of data focusing on key aspects of this new paradigm: (1) implementation of the QbD concept and associated regulatory frameworks (ICH Q8-Q10), (2) optimization and scale-up of critical processes such as microfluidic mixing, and (3) addressing platform-specific challenges related to product performance (e.g., size control and PDI for LNPs).

Transitioning from design to a controlled manufacturing strategy. A synthesis of current research on microfluidic methods shows that key process parameters, such as flow rate ratio (FRR) and total flow rate (TFR), directly determine critical quality attributes of liposomes and LNPs, primarily size and polydispersity [8,16,64]. This approach essentially puts Quality by Design (QbD) principles into practice, ensuring reproducibility and control at the mixing, dilution, and purification stages.

Platform specifics: LNPs and liposomes. For LNPs, the micromixing process is key, where the flow rate ratio (FRR) directly determines size and polydispersity, which is critical for reproducibility and scalability when using continuous microfluidic systems [64]. The final formation of critical quality parameters occurs during the dilution and purification steps [65]. For liposomes, microfluidic methods ensure controlled particle formation with a narrow size distribution, complementing traditional approaches [8].

Size and stability control. Research in the field of microfluidic synthesis highlights that precise control of liposome characteristics (size, composition, encapsulation efficiency) is crucial for their translational potential and meets the stringent standards required for pharmaceutical products. The efficacy and biodistribution of LNPs are highly dependent on particle size. The optimal range (~60–120 nm) provides a balance between activity and clearance, while precision synthesis of ultrasmall LNPs (<50 nm) can improve liver delivery efficiency [16]. Tuning of process parameters (solvent fraction, mixing energy) is necessary to prevent aggregation or excessive size reduction during scale-up, which requires in vivo validation [64,66].

Scale-up and tech transfer. Scaling strategies range from increasing the number of microreactors to designing systems with larger channels while maintaining key mixing parameters and creating continuous lines with integrated purification steps [8]. Successful technology transfer requires strict consistency of input materials (lipids, nucleic acids, buffers) and process validation within the established parameter space. Typical issues—fluctuations in solvent composition, membrane fouling, and residence time changes affecting encapsulation efficiency—should be included in risk assessments and monitored throughout the process [65].

Similarly, for polymeric nanocarriers, the transition from batch to fully continuous, integrated manufacturing processes has been demonstrated to address batch-to-batch variability and scalability challenges, achieving high-throughput GMP-compliant production [22].

Regulatory and analytical expectations. Successful translation requires comprehensive chemistry, manufacturing, and control (CMC) data, as supported by studies demonstrating the importance of controlling the size, purity, and stability of nanoparticles for their preclinical and clinical efficacy [8,16,20].

Thus, the synthesis of manufacturing research covered in this review highlights a critical evolution: modern process control, based on QbD (Quality by Design) principles, has ceased to be a secondary concern and has become a primary factor determining translational success. The data presented confirm that scalable and reproducible manufacturing, enabled by technologies such as microfluidics and continuous processes, is inseparable from the initial therapeutic design. It forms the crucial bridge between preclinical promise and clinical reality for complex drug delivery systems. Furthermore, digital tools such as predictive kinetic models can forecast critical quality attribute (CQA) loss of mRNA vaccines based on temperature monitoring data, offering a solution to cold-chain logistics challenges [35].

### 3.4. Synthesis of Safety, Immunogenicity, and Translational Evaluation Data

The transition from preclinical models to clinical application of nanodelivery systems requires a thorough reassessment of safety paradigms. A synthesis of studies from 2016 to 2025 demonstrates that these platforms can induce class-specific adverse effects that are often undetected by traditional testing protocols developed for small-molecule drugs. These effects arise from complex interactions between the synthetic nanocarrier surface and biological systems—from immediate protein corona formation to delayed immune responses. This section summarizes current evidence and approaches to identifying and mitigating key risks associated with excipient immunogenicity (e.g., anti-PEG reactions), hemocompatibility, and neurotoxicity during targeted delivery to the central nervous system. This section also highlights the need for specialized preclinical models that adequately predict clinical safety.

A finding from the reviewed translational studies is that nanocarriers can trigger class-specific adverse responses that are seldom seen with conventional small-molecule formulations. A prominent example, highlighted across multiple reports, is the induction of systemic inflammatory and hypersensitivity reactions. For instance, the systemic administration of potent immunostimulators formulated in nanocarriers can lead to severe, dose-limiting toxicities [67]. This underscores a key principle emerging from the data: formulation strategies must be designed to localize the immune response and minimize systemic exposure. Separately, and with growing clinical relevance, immunogenicity to excipients—most notably anti-poly(ethylene glycol) (anti-PEG) antibodies—which can both reduce exposure (via accelerated blood clearance) and, in rare patients, precipitate immediate hypersensitivity reactions; risk correlates with PEG molecular weight, presentation (free vs. grafted), and prior exposures in drugs/cosmetics [68]. The development of new platforms such as ionizable polymeric micelles aims to overcome this limitation, demonstrating reduced ABC effect and immunogenicity compared to LNPs in preclinical models [69].

A risk-based, material-aware approach is paramount for safety assessment. The analysis of included studies confirms that nanocarrier formulation attributes (size distribution, surface charge/chemistry, release profile) directly influence the strategy for their preclinical evaluation and benefit-risk analysis. For example, MacCuaig et al. (2022) examined in detail how the size and dose of mesoporous silica nanoparticles affect the acute toxicity profile following intravenous administration, illustrating the need for materials-aware safety assessment [70]. Furthermore, assessing the immunotoxicity of nanomedicines requires specialized approaches beyond standard protocols. Lu et al. (2019), when developing a TLR7/8 agonist-based immunomodulator, conducted targeted analyses (including cytokine profiling) outside standard toxicology packages, which was critical for identifying platform-specific, dose-limiting systemic toxicity [67]. Because conventional in vitro tests often mispredict in vivo outcomes for nanosystems, studies show that the formation of a protein corona can critically alter cellular interactions and must be accounted for in test design [39]. Moreover, studies demonstrate that in vivo toxicity of nanomaterials such as graphene oxide nanosheets is determined not by their intrinsic properties but by the specific composition of the adsorbed protein corona (e.g., complement component C3) and the immune status of the host organism, highlighting the need for personalized approaches to safety assessment [71].

Furthermore, an analysis of hemocompatibility studies confirms its critical importance for parenteral delivery systems. Experimental studies demonstrate that testing conditions (e.g., the use of whole human blood and physiologically realistic shear conditions) significantly impact the results of hemolysis, complement activation, coagulation, and platelet adhesion assessments. In particular, Blok et al. (2019) demonstrated that an incubation time of 60 min is optimal for differentiating hemocompatible from incompatible materials in a dynamic model, while Sperling et al. (2023) found a dependence of platelet activation and hemolysis on shear rate in a new incubation system [72,73]. While surface hydration and near-neutral/zwitterionic coronas generally reduce biofouling and thrombogenicity, performance remains device- and context-dependent. For CNS-directed delivery via receptor-mediated transcytosis—particularly transferrin-receptor (TfR) pathways—safety and translation hinge on controlled receptor occupancy, peripheral ‘sink’ effects in TfR-rich tissues, and trafficking fate (recycling versus lysosomal routing); ligand affinity/valency must be tuned (often moderate-affinity, monovalent or pH-labile formats) to favor productive transcytosis over endothelial retention and to avoid barrier saturation [61]. Finally, case reports and cohort studies document rare, IgE-mediated anaphylaxis to PEG (and cross-reactivity with polysorbates), underscoring the need for careful history, labeling, and (when justified) excipient substitutions or desensitization strategies in at-risk patients [68].

Taken together, the safety data synthesis highlights that the immune system is the primary arbiter of the fate and tolerability of nanoscale delivery systems in vivo. Standard protocols that fail to account for phenomena such as protein corona dynamics, preformed immunity to excipients, or specific interactions with blood cells during physiological changes lead to false-positive or false-negative results. Therefore, translationally relevant safety assessments must be material-aware and immunology-aware, integrating specialized immunogenicity and hemocompatibility testing into the earliest stages of development. Only such a proactive approach allows for the identification of platform-specific risks prior to clinical trials and lays the foundation for a favorable benefit-risk profile, necessary for the approval of innovative therapeutics.

### 3.5. Synthesis of Clinical Landscape: Approved Systems and Promising Candidates in Development

Clinical success and failure serve as the final arbiter for all engineering strategies discussed in the previous sections. Analysis of approved products and late-stage candidates allows us to formulate fundamental principles for successful translation. The key conclusion emerging from this synthesis is that long-term clinical and commercial success is achieved not by the platform’s versatility, but by its precise alignment with a specific therapeutic objective and disease biology. Below, we analyze how this principle is being implemented in practice: from liposomal anthracyclines and LNPs for RNA therapy to innovations in microneedles and biomimetics, which are on the verge of clinical implementation.

Clinical validation of nanotherapeutics, beginning with liposomal doxorubicin (Doxil^®^), has identified two fundamental principles for success. First, matching the platform to the disease biology is key. For example, pegylated liposomes (PLD, Doxil^®^/Caelyx^®^) provide prolonged tumor exposure, which increases efficacy and reduces cardiotoxicity compared to free doxorubicin [74]. Similarly, liposomal amphotericin B (AmBisome) dramatically reduces nephrotoxicity while maintaining fungicidal activity [75]. LNPs, leveraging their natural tropism for hepatocytes, have become the basis for siRNA therapies (patisiran) and mRNA vaccines, providing potent therapeutic effects and the potential for rapid global deployment [11,12].

Secondly, success is determined by rigorous discipline in the field of CMC. Products with reproducible characteristics (size, PDI, encapsulation efficiency) demonstrate more predictable pharmacokinetics and simplify technology transfer (see Section 3.3). Despite remaining challenges—immunogenicity (e.g., reactions to PEG) [68], scalability difficulties [16], and patient heterogeneity—technological advances (new stealth coatings, ligands, microfluidic manufacturing) are improving clinical reproducibility [38,76]. The most significant approved delivery systems and their key clinical benefits are summarized in Table 1.

Beyond commercially approved formulations, several innovative delivery platforms have demonstrated compelling proof-of-concept in advanced clinical trials, signaling the next wave of translational progress (Table 2; see Supplementary File S4 for the complete list of promising systems, based on a synthesis of studies). This pipeline showcases a strategic expansion into new administration routes (e.g., transdermal, intranasal) and more sophisticated targeting mechanisms. Notably, a dissolving microneedle patch (MNP) for influenza vaccination successfully completed a Phase 1 trial, demonstrating safety and immunogenicity comparable to the standard intramuscular injection while offering a markedly improved patient experience and potential for self-administration [30]. This milestone represents a critical step towards pain-free, logistically simplified vaccination programs.

Simultaneously, novel formulation strategies are pushing the boundaries of efficacy in preclinical models. For instance, bilosome-based mucoadhesive in situ gels have demonstrated remarkable preclinical efficacy, increasing the brain bioavailability of an anti-migraine drug by over 10-fold compared to intravenous administration and achieving a ~98% direct nose-to-brain transport percentage in rats, showcasing a potent strategy to overcome nasal clearance [31].

In conclusion, the clinical landscape of drug delivery is characterized by a dynamic continuum from validated platforms to emerging paradigms. Approved systems like PLD, LNPs, and ADCs have cemented the principle that carrier design must be dictated by disease biology—whether leveraging passive accumulation, inherent tissue tropism, or molecular targeting. The next wave of candidates, from patient-centric microneedles to intelligent biomimetic systems, builds upon this foundation while introducing new dimensions of spatial control, adaptive release, and immune stealth. The collective evidence from both approved and late-stage candidates underscores that the most significant translational leap occurs not merely from a novel material, but from its strategic application to an unmet clinical need, guided by a deep integration of engineering, biological, and patient-centered considerations.

### 3.6. Synthesis of Translational Hurdles: Cost, Scalability, and Accessibility

Following the engineering challenges of biological barriers (Section 3.2) and reproducible manufacturing (Section 3.3), the ultimate determinant of a technology’s real-world impact is its economic viability and equitable access. The analysis of contemporary studies shows that the translational gap between a promising laboratory prototype and a widely available therapeutic is often defined not by scientific feasibility, but by socio-economic and infrastructural constraints. Consequently, the real-world adoption of advanced nanomedicines is critically dependent on overcoming hurdles related to cost, scalability, and global accessibility [77,78]. A comparative analysis of platform technologies reveals significant disparities in their cost structures and industrial scalability, which ultimately dictate their clinical and commercial trajectory.

This analysis uncovers a persistent translational paradox: there is a clear contradiction between the functional complexity of DDS and their economic and industrial feasibility. Systems with complex structures and high functional potential, such as dendrimers and natural EVs, currently demonstrate low cost-effectiveness and significant difficulties in industrial scaling [21]. In contrast, LNPs and liposomes, which have achieved the greatest clinical and commercial success, are based on simpler principles of structural organization and are characterized by highly reproducible, proven processes for large-scale production [20].

This analysis reveals that the primary barriers are not only technical but economic. The high capital investment required for advanced manufacturing technologies, coupled with the need for specialized infrastructure, creates a significant entry barrier, particularly for small and medium-sized enterprises. These cost factors have direct implications for global accessibility. For instance, the cold-chain requirements for LNP-based vaccines (where the stability of the mRNA is dependent on strict, unbroken temperature control [79]) create persistent barriers to equitable access in low- and middle-income countries [33]. To address this critical limitation, research is actively developing thermostable LNP-mRNA formulations that maintain efficacy after storage at room temperature, as demonstrated in preclinical models [17]. The combination of production challenges and complex logistics has become apparent during the COVID-19 pandemic, further exacerbating global inequalities in access [78].

New manufacturing paradigms are particularly important for breaking this impasse related to cost and scalability. For lipid-based systems, microfluidic platforms are proving to be an optimal solution for controlled and scalable synthesis [8]. This precise control directly translates to enhanced in vivo performance, as demonstrated by the synthesis of ultra-small, highly potent LNPs for siRNA delivery [16]. Similarly, advanced and scalable manufacturing processes are being developed to improve the reproducibility and reduce costs of polymeric and lipid-based systems, as demonstrated for continuous production of PLGA nanoparticles [20] and scaled-up microfluidic synthesis of nanolipomers [65].

**Figure 2:**
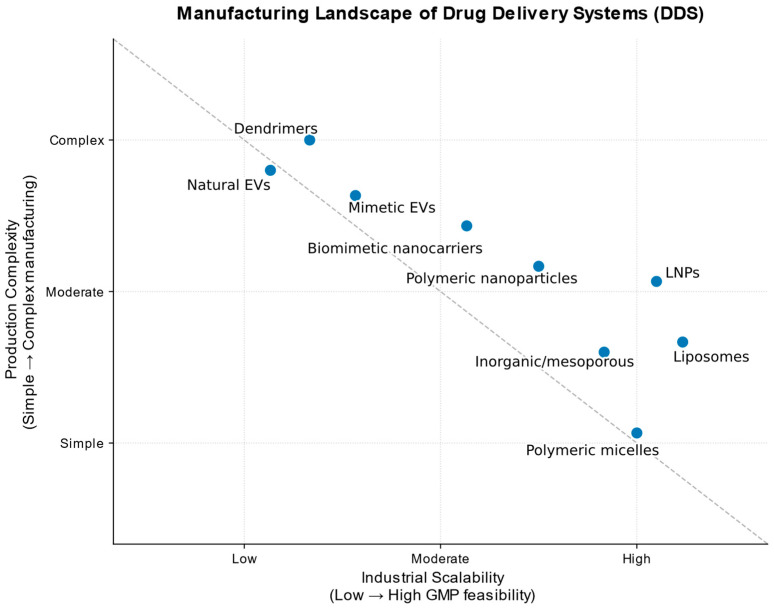
Conceptual positioning of major drug delivery platforms based on their relative industrial scalability and manufacturing complexity, as informed by primary research on platform-specific production challenges and scale-up studies [8,16,20,23,76,80,81,82]. The diagram reflects qualitative trends derived from reported studies rather than quantitative economic metrics. Relative manufacturing complexity does not directly correspond to material cost. The scheme does not imply differences in therapeutic efficacy or clinical performance.

From a translational perspective, manufacturing complexity and industrial scalability remain critical determinants of clinical feasibility across drug delivery platforms, as summarized in Figure 2.

Data analysis confirms the existence of a key translational paradox: the maximum functional complexity and biomimetic sophistication of a platform are often inversely proportional to its chances of becoming a widely available drug. The solution to this paradox lies in the field of targeted engineering, where perfection is sacrificed for reproducibility, and versatility is sacrificed for specialization for a specific, scalable manufacturing task. Further progress will depend on the convergence of three previously disparate fields—pharmaceutical engineering, health economics, and global logistics—so that innovations in drug delivery are measured not only by patents but also by the number of patients who can afford them.

### 3.7. Future Directions and Emerging Paradigms

Building on established principles and identified translational challenges, the field of drug delivery is undergoing a profound conceptual transformation. A synthesis of contemporary research points to the emergence of a new generation of systems that are no longer simply passive carriers, but rather adaptive, communicative, and intelligent therapeutic agents. This section examines several interconnected paradigms defining this frontier: natural delivery vectors (extracellular vesicles), bioinspired designs blurring the line between synthetic and living substances, digital manufacturing of responsive dosage forms, platforms for delivering the most complex biological payloads (e.g., genome editors), and the integration of artificial intelligence to guide both design and autonomous function. Together, these trends shape a vision of a future in which drug delivery becomes personalized, predictive, and seamlessly integrated with patient physiology.

#### 3.7.1. Extracellular Vesicles (EVs) as Natural Delivery Vectors

The synthesis of current data allows us to consider EVs not simply as biological carriers but as an evolutionarily optimized therapeutic platform whose potential stems from fundamental biological properties: biocompatibility, inherent tropism, and mechanisms for efficient cytosolic release. These properties provide EVs with an excellent ability to overcome critical biological barriers (such as the BBB and dense tumor stroma), which remain challenging for many synthetic analogues. These inherent advantages are supported by comparative studies. For instance, tumor-derived EV-hybrid nanovesicles enhanced siRNA delivery and tumor-homing compared to standard liposomes [83], while cardiac progenitor EVs delivered *VEGF-A* mRNA to ischemic heart tissue more effectively and with lower immunogenicity than clinical-grade LNPs [84]. A quantitative comparison has shown that EVs can deliver siRNA with an efficiency several orders of magnitude higher than that of standard synthetic nanoparticles [85].

In a myocardial ischemia model, EVs demonstrated highly efficient delivery of therapeutic *VEGF-A* mRNA with significantly lower immunogenicity than clinical LNPs [84]. The potential of EVs is also being unlocked through hybrid approaches: the creation of hybrid nanovesicles based on EV membranes significantly improved tumor tropism and siRNA delivery compared to liposomes [83], while an innovative two-step strategy (LNPs transfect cells for subsequent secretion of therapeutic EVs) highlighted the high functional efficacy and biocompatibility of the produced EVs [86]. Mechanistic insights into EV-liposome interactions further support this direction. For instance, Fisher et al. (2025) demonstrated that acidic conditions, mimicking the tumor microenvironment, promote clustering of cancer-derived EVs and enhance their fusion with liposomes, suggesting a promising mechanism for designing environment-responsive hybrid delivery systems [87]. This emerging consensus clearly indicates that EVs offer a solution to key challenges of synthetic delivery systems, particularly in terms of targeting specificity and minimizing adverse immune responses.

However, a clear translational paradox exists: the high biological activity of EVs is coupled with difficulties in standardizing their production, which directly impacts the reproducibility of results [80,81]. This determines the strategic choice: EVs are optimal for therapies requiring maximum specificity (e.g., neurodegenerative diseases), while scalable synthetic systems retain an advantage for mass use (vaccination) [88].

Overcoming this paradox lies not in simplifying EVs, but in their targeted engineering—transforming them from a natural product into a programmable vector, for example, through genetic modification of producer cells [82]. Thus, EVs occupy a unique niche for the creation of highly personalized therapies, where their biological complexity becomes a key advantage.

#### 3.7.2. Biomimetic and Cell-Membrane–Coated Drug-Delivery Platforms

Biomimetic delivery systems based on cell membrane shells allow synthetic nanoparticles to mimic natural cells. By coating the particle core with a membrane derived from red blood cells, white blood cells, or tumor cells, the system inherits a complex of surface proteins and signaling motifs that recreate key physiological interactions [79]. This provides cell-type-specific advantages: red blood cell membranes carry “individual” markers (e.g., CD47) to evade the immune system and prolong circulation; white blood cell membranes use adhesion receptors to target inflamed endothelium [59]; and tumor cell membranes provide homotypic recognition for tumor accumulation [79]. Thus, in preclinical models, this biomimetic approach has demonstrated superior biodistribution and targeting specificity compared to traditional ligand-based strategies.

Despite these promising biological benefits, significant translational hurdles must be overcome. Key challenges include preserving membrane integrity and protein function during manufacturing, controlling batch-to-batch variability due to the heterogeneity of biological source materials, and rigorously evaluating long-term in vivo stability and immunogenicity. Addressing these issues is essential for progressing these platforms from proof-of-concept to clinical application.

In parallel, even more integrated approaches are developing, blurring the line between synthetic carriers and living cells. Cell surface engineering technologies, such as the Cellnex platform, enable the deposition of multifunctional nanocomplexes directly onto the surface of living therapeutic cells (e.g., T cells, macrophages, or erythrocytes). This transforms the cells themselves into programmable hybrid systems that retain natural functions (targeting, circulation) but acquire enhanced properties for delivery or immunotherapy, as demonstrated in preclinical models [33].

#### 3.7.3. 3D/4D-Printed Dosage Forms and Adaptive Material Architectures

Additive manufacturing has rapidly evolved from a niche engineering tool into a transformative approach for personalized pharmaceutics. Three-dimensional (3D) printing enables precise spatial placement of active pharmaceutical ingredients within complex geometries, allowing the fabrication of dosage forms tailored to individual pharmacokinetic requirements [89]. By adjusting parameters such as pore size, internal architecture, and material composition, printed systems can modulate dissolution rates and deliver complex drug combinations [90].

Four-dimensional (4D) printing enables drug formulations to undergo predictable shape or property changes after administration in response to physiological stimuli like pH, allowing for sophisticated functions such as site-specific drug release [91]. Advances in polymer nanocomposites are improving the robustness of these printed structures. This tunability is key for 4D systems, enabling dynamic, long-term drug release modulation. For instance, shape-memory polymer composites (SMPCs) can be programmed for targeted deployment and release drugs in response to triggers like heat or magnetic fields. Incorporating nanomaterials like nanocellulose or Fe_3_O_4_ nanoparticles further refines this responsive control [92].

Despite progress, 3D/4D printing faces significant translational hurdles. Variability in polymer feedstock properties directly impacts critical quality attributes, such as drug content uniformity and dissolution profiles in printed tablets [90]. This, along with challenges related to print-path fidelity and the thermal sensitivity of APIs, complicates reproducibility. For widespread clinical implementation, these fundamental issues of materials science and process reproducibility need to be addressed. Nonetheless, the technology’s core advantage lies in enabling personalized polypills with adaptable dosages and release kinetics, which can significantly improve patient adherence.

Through these developments, 3D and 4D printing are emerging as powerful technologies capable of aligning drug delivery with individual patient physiology, thereby addressing key reviewer concerns regarding stability, release kinetics, and real-world applicability.

#### 3.7.4. Delivery of Genome-Editing Tools and Complex Biologics

Genome-editing therapeutics, including CRISPR–Cas nucleases, base editors, and prime editors, impose delivery requirements that are substantially more demanding than those for conventional nucleic-acid medicines. Their biological activity depends on precise intracellular trafficking, efficient nuclear localization, and tightly controlled exposure windows, since prolonged nuclease presence increases the risk of off-target DNA modifications and immunogenic responses. Compared with siRNA or mRNA payloads, genome-editing complexes are larger, structurally more rigid, and inherently less permeable across cellular and endosomal membranes, making carrier design a central determinant of therapeutic success.

LNPs remain the most clinically validated vehicles for in vivo gene editing, particularly in the liver. Research into alternative platforms, including polymer-based systems, aims to overcome this hepatotropic bias and enable editing in extrahepatic tissues. The work by Rosenblum et al., demonstrates that CRISPR-Cas9 mRNA, delivered via targeted LNPs, can achieve efficient gene editing in tumor cells in vivo (e.g., glioblastoma and ovarian cancer models), leading to significant tumor growth inhibition and extended survival, largely due to the ionizable lipids facilitating endosomal escape [93]. However, the reliance of LNPs on hepatotropic biodistribution restricts applications largely to hepatic targets. To circumvent this limitation and achieve a more controllable therapeutic window, direct delivery of ribonucleoprotein (RNP) complexes provides a transient and highly controllable editing window, mitigating long-term nuclease expression and reducing the likelihood of immunological activation and unintended genomic alterations [94]. Engineered gold nanoparticles (AuNPs) have been successfully used for direct cytosolic delivery of Cas9-RNP in vitro, achieving high editing efficiency while bypassing endosomal entrapment [95]. Despite these advantages, RNP formulations face additional challenges related to protein stability, cytosolic release, and rapid clearance from circulation.

A key barrier to the clinical translation of genome-editing tools is the prevalent pre-existing immunity to Cas proteins in humans, which can cause rapid clearance and inflammatory reactions [96]. Efficient delivery to extrahepatic tissues (e.g., muscle, CNS, lung) remains a major challenge, demanding new material and targeting strategies. Engineering the cargo itself, such as optimizing the physicochemical properties of the Cas9 RNP complex, represents a promising approach to enhance delivery efficiency and editing specificity [97]. Overcoming these mechanistic hurdles is crucial for the safe and responsible deployment of these therapies.

#### 3.7.5. AI-Guided and Autonomous Intelligent Delivery Systems

Machine learning (ML) models trained on large datasets of nanoparticle physicochemical properties are enabling increasingly accurate and mechanistically informed predictions of their biological fate and safety. This predictive power spans multiple levels: from forecasting the toxicity of inorganic nanoparticles through models based on their dissolution kinetics [98], to enabling in silico screening of polymeric gene carriers by predicting transfection efficiency and cytotoxicity directly from chemical structure [99]. Furthermore, ML models can elucidate intracellular mechanisms, such as using the lysosomal dissociation degree as a novel descriptor to accurately predict the toxicity of metal oxide nanoparticles in immune cells [100]. Collectively, these approaches are shifting nanocarrier design from empirical screening to rational, data-driven optimization.

The next frontier in drug delivery is the development of autonomous, closed-loop systems. These “intelligent” carriers go beyond passive transport, incorporating molecular logic to sense, process, and respond to specific biological signals—such as elevated ROS, abnormal enzyme activity, or tumor-specific pH [101,102]. Using multi-input logic gates (e.g., an AND-gate that activates a prodrug only after a tumor receives a specific enzyme), they achieve precise, conditional drug release exclusively within pathological microenvironments.

This paradigm shift from static vehicles to dynamic therapeutic modules enables treatments that could autonomously adjust dosing, release profiles, or drug combinations in real-time based on disease progression. This aligns drug delivery with the ultimate goals of personalized and adaptive medicine.

Taken together, the new paradigms discussed outline the next, “smart” phase of the evolution of drug delivery systems, where biological understanding, precision materials science, and digital technologies converge into a single engineering discipline. A key distinction of this stage is the transition from the creation of static carriers to the design of dynamic, context-sensitive therapeutic systems capable of sensing, processing biological signals, and adapting in vivo. Although each of these platforms—from programmable extracellular vesicles to autonomous intelligent systems—is at different stages of maturity, they share a common goal: to overcome the fundamental limitations of traditional nanomedicine in terms of specificity, temporal control, and learning ability. Successfully translating these ambitious concepts into the clinic will require an unprecedented level of interdisciplinary collaboration and new regulatory flexibility. However, it is precisely they that are ultimately capable of realizing the ultimate goal of personalized medicine: providing the right drug in the right place, at the right time, and in the right dose, determined not by an empirical protocol, but by the patient’s current condition.

## 4. Discussion

### 4.1. Brief Summary of the Main Findings

A systematic review of data from 2016 to 2025 allows us to synthesize several fundamental principles guiding the modern translation of drug delivery systems (DDSs).

**Figure 3:**
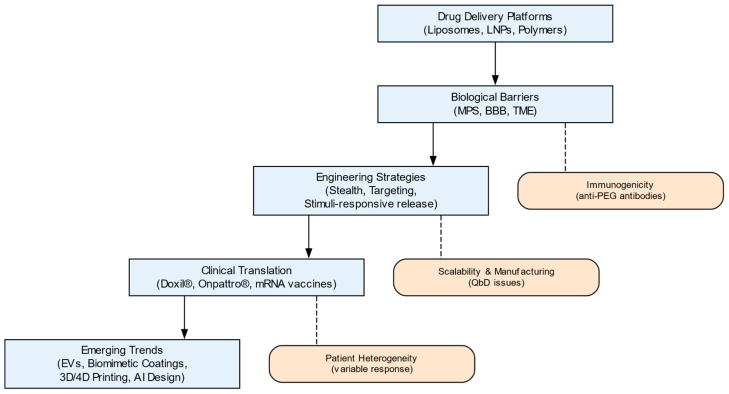
Roadmap of advanced drug delivery systems (DDS): key stages, barriers, and emerging trends. Classical drug delivery platforms evolved through engineering strategies and translational milestones toward next-generation biomimetic and intelligent systems. Figure created using Graphviz (https://graphviz.org, accessed on 5 November 2025).

The synthesis of evidence highlights that while established platforms have achieved clinical success, their translation is constrained by persistent barriers. This discussion explores how emerging technological directions may address these limitations and shape the future of the field. Recent progress in nanocarrier design has been shaped by both biological barriers and translational challenges (see Figure 3).

As illustrated in Figure 3, several core principles emerge from this evolutionary analysis. First, clinical success directly depends on the precise matching of the physicochemical properties of the nanocarrier (size, surface chemistry, mechanical properties) with the specific biological barriers of the target disease, whether this be tumor stromal density, blood–brain barrier selectivity, or mucociliary clearance. Second, engineering strategies for overcoming these barriers—from stealth coatings to receptor-mediated targeting—must be developed taking into account their dynamic interaction with the biological environment, primarily the developing protein corona. Third, a key translational paradox has been identified: the gap between the laboratory efficacy of complex platforms (dendrimers, extracellular vesicles) and their clinical availability is due not to biological but to economic and technological limitations—high costs and low scalability of production. Finally, the field is moving toward the creation of adaptive, intelligent systems capable of signal sensing and spatiotemporal release control (biomimetic carriers, 4D printing, AI-optimized designs).

### 4.2. Interpretation and Significance

These findings mark a paradigm shift in approaches to DDS development. While early nanomedicine emphasized passive accumulation in pathological tissues (EPR effect) and maximizing drug loading, the current paradigm requires holistic “barrier-to-barrier design.” This means that a successful platform must be designed not as a static structure, but as a dynamic entity whose in vivo behavior is predicted based on interactions with the protein corona, the immune system, and the physiological characteristics of the route of administration. The identified priority of reproducible and economically viable production over maximum functional complexity indicates a crucial shift: the focus of translational research is now not only on “can it work?” but also on “can it be reproducibly and affordably manufactured?” This elevates questions of health economics and global equity to the level of fundamental scientific limitations.

### 4.3. Comparison with Existing Literature

The findings of this review are generally consistent with recent narrative reviews, which also highlight a paradigm shift toward engineering delivery systems capable of overcoming biological barriers (such as BBB and tumor microenvironment) while simultaneously addressing scalability, manufacturability, and regulatory pathways to enable clinical translation [103,104]. However, the present study makes a clarifying contribution. First, we confirm and elaborate on the thesis regarding the critical role of the protein corona by systematizing data demonstrating that it cannot only prolong circulation but also completely negate the active targeting function in vivo [61], necessitating a reconsideration of the design of ligand-modified systems. Second, unlike many reviews optimistically describing the potential of EVs, our synthesis clearly articulates their inherent translational paradox—the inverse relationship between outstanding biological efficacy and the technological immaturity of standardized manufacturing—proposing engineering as a way to resolve it. Third, we expand the discussion on routes of administration by emphasizing the inextricable link between nanoformulation design and delivery device co-engineering to achieve optimal therapeutic outcomes.

### 4.4. Limitations of the Review

A number of limitations should be considered when interpreting the presented findings. Despite a rigorous systematic search, significant methodological heterogeneity among the included studies (various animal models, characterization protocols, and efficacy criteria) precluded a formal meta-analysis and required a narrative synthesis, which may introduce a subjective component. The review focused on English-language publications, which may have biased studies from certain regions. Furthermore, for the fastest-growing areas (e.g., CRISPR delivery or AI applications), the evidence base for the period 2016–2025 is still evolving and is characterized by a predominance of preclinical and proof-of-concept studies, making conclusions on these topics more of a trend indicator than a definitive principle. These limitations highlight the need for further standardized studies and clinical validation.

## 5. Conclusions and Directions for Future Research

In conclusion, this review consolidates the current evidence guiding the path from laboratory prototype to clinically relevant therapeutics. A fundamental finding is the need for an integrative approach in which biological design, manufacturing processes, and economic viability assessment are considered as interrelated elements from the very beginning of platform development.

Based on this synthesis, the following priority areas for future research can be identified:

1. Development of “corona-aware” design: Creation of standardized in vitro and in silico models for predicting the formation and evolution of protein coronas, which will enable prediction of the fate of nanocarriers in vivo and optimization of targeting strategies.
2. Engineering for reproducibility and accessibility: Actively developing and validating continuous, cost-effective manufacturing processes (e.g., microfluidics-based) for promising but complex platforms such as EVs and biomimetic systems, incorporating Quality by Design (QbD) principles.
3. Creating new preclinical models: Transitioning from simple to more complex models that account for disease heterogeneity (e.g., tumor stromal heterogeneity) and the human immune response, for a more accurate assessment of translational potential.
4. Integrating digital tools into the full cycle: Extensive use of artificial intelligence and machine learning not only for in silico screening of materials but also for optimizing manufacturing processes, managing product stability (e.g., predictive models for the cold chain), and designing adaptive, closed-loop control systems.

The implementation of this research agenda, based on interdisciplinary collaboration, will contribute to the transformation of advanced delivery systems from a tool of fundamental science into the cornerstone of personalized, precise, and accessible medicine of the future.

## Figures and Tables

**Table 1:** Representative approved DDS and headline clinical advantages.

Product (Approval Year)	Carrier	Indication(s)	Headline Clinical Benefit
Doxil^®^/Caelyx^®^ (1995 EU/1999 US)	PEGylated liposome (PLD)	Kaposi’s sarcoma, ovarian cancer, multiple myeloma	Reduced cardiotoxicity and sustained tumor exposure vs. conventional doxorubicin.
AmBisome^®^ (1997)	Liposomal amphotericin B	Invasive fungal infections	Markedly reduced nephrotoxicity with maintained fungicidal efficacy.
Abraxane^®^ (2005)	Albumin-bound paclitaxel (nab-paclitaxel)	Metastatic breast cancer; later pancreas, NSCLC	Improved tolerability and response rates via solvent-free, targeted delivery.
Onpattro^®^ (2018)	LNPs (siRNA, patisiran)	Hereditary transthyretin (hATTR) polyneuropathy	Robust target silencing with meaningful improvement in neuropathy and QoL.
Comirnaty^®^ (2020)	LNPs (mRNA)	COVID-19 prevention	High efficacy in phase III; rapid global scale-up and deployment
Spikevax^®^ (2021)	LNPs (mRNA)	COVID-19 prevention	Strong immunogenicity; flexible adaptation to variants
Vyxeos^®^ (2017)	Liposomal daunorubicin/cytarabine	High-risk AML	Improved overall survival vs. standard 7 + 3 regimen
Marqibo^®^ (2012)	Liposomal vincristine	Ph-negative ALL	Enables higher effective dose; prolonged circulation; reduced neurotoxicity
DepoCyt^®^/DepoCyte^®^ (1999)	DepoFoam liposomal formulation	Lymphomatous meningitis	Extended CSF exposure; reduced need for frequent lumbar punctures
Visudyne^®^ (2000)	Liposomal verteporfin	Age-related macular degeneration (AMD)	Targeted photodynamic action with lower systemic toxicity
Adcetris^®^ (2011)	Antibody–drug conjugate (ADC, brentuximab vedotin)	Hodgkin lymphoma; ALCL	Selective CD30 targeting enhances efficacy and reduces off-target effects.
Enhertu^®^ (2019)	ADC (trastuzumab deruxtecan)	HER2-positive metastatic breast cancer	High response rates via potent, targeted payload delivery.
Susvimo^®^ (Port Delivery System with ranibizumab) (2021)	Refillable intravitreal implant	Neovascular (wet) age-related macular degeneration (nAMD)	Continuous drug delivery extends treatment intervals to 6 months, preferred by patients [29].

**Table 2:** Promising drug delivery platforms in advanced clinical development.

Platform/Candidate	Clinical Stage	Key Innovation/Outcome	Reference
Influenza Vaccine Microneedle Patch (TIV-MNP)	Phase 1 Trial	Safe, immunogenic, patient-friendly alternative to intramuscular injection.	[30]
Bilosomal Mucoadhesive In situ Gel (Zolmitriptan)	Preclinical (In vivo PK in rodents)	>10-fold increase in brain bioavailability via direct nose-to-brain transport (~98%).	[31]
Albumin-PEG NPs with Permeation Enhancer (Bevacizumab)	Preclinical (In vivo, rats)	Enabled oral bioavailability of a monoclonal antibody (~3.7%, 1000-fold increase).	[32]
“Cellnex” Cell Surface Engineering	Preclinical (In vivo proof-of-concept)	Coats living cells with nanocomplexes, enhancing targeting (11-fold lung delivery) and immunotherapy.	[33]
Tunable Leukocyte-Membrane Coated NPs	Preclinical (In vivo, inflammation models)	Quantitative tuning of protein–lipid ratio in coatings enhances targeting to inflamed endothelium.	[34]
Predictive Stability Model for LNP-mRNA Vaccines	In silico/Translational Solution	Kinetic model predicts CQA loss from temp. data, addressing cold-chain barrier.	[35]

## Data Availability

The original contributions presented in this study are included in the article/Supplementary Material. Further inquiries can be directed to the corresponding authors.

## Supplementary Materials

The following supporting information can be downloaded at: https://www.mdpi.com/article/10.3390/pharmaceutics18010011/s1, File S1: Systematic Review Protocol; File S2: Full Electronic Search Strategy; File S3: Data Extraction Table; File S4: Promising drug delivery platforms in advanced clinical development.

## Abbreviations

The following abbreviations are used in this manuscript:

ABC	Accelerated Blood Clearance
AI	Artificial Intelligence
BBB	Blood–Brain Barrier
CARPA	Complement Activation-Related Pseudoallergy
CMC	Chemistry, Manufacturing, and Controls
CQA	Critical Quality Attribute
CRISPR	Clustered Regularly Interspaced Short Palindromic Repeats
DDS	Drug Delivery System(s)
DLS	Dynamic Light Scattering
DSPC	Distearoylphosphatidylcholine
EMA	European Medicines Agency
EPR	Enhanced Permeability and Retention
EV	Extracellular Vesicle
FDA	Food and Drug Administration
FRR	Flow-Rate Ratio
GC	Gas Chromatography
HPLC	High Performance Liquid Chromatography
ICH	International Council for Harmonization
LNPs	Lipid Nanoparticles
ML	Machine learning
MMAD	Mass Median Aerodynamic Diameter
MPS	Mononuclear Phagocyte System
MSN	Mesoporous Silica Nanoparticle
NMR	Nuclear Magnetic Resonance
NP	Nanoparticle
PCL	Poly(ε-caprolactone)
PDI	Polydispersity Index
PEG	Poly(Ethylene Glycol)
PLA	Polylactic Acid
PLD	Pegylated Liposomal Doxorubicin
PLGA	Poly(Lactic-co-Glycolic Acid)
PQS	Pharmaceutical Quality System
QbD	Quality by Design
QTPP	Quality Target Product Profile
RES	Reticuloendothelial System
RNP	Ribonucleoprotein
SAXS	Small-Angle X-ray Scattering
SEC	Size Exclusion Chromatography
TfR	Transferrin Receptor
TFR	Total Flow Rate
XPS	X-ray Photoelectron Spectroscopy
