# Supplementary material for: A Systematic Review of Advanced Drug Delivery Systems: Engineering Strategies, Barrier Penetration, and Clinical Progress (2016–April 2025)
Source: Pharmaceutics. 2025 Dec 22;18(1):11. doi: 10.3390/pharmaceutics18010011 (PMC12845006; doi:10.3390/pharmaceutics18010011)
Supplement: Supplementary file 1 [file pharmaceutics-18-00011-s001.zip › Supplementary File S1 Systematic Review Protocol.pdf]

Supplementary File S1

PROTOCOL: A systematic review of advanced drug delivery systems: engineering strategies, barrier penetration, and clinical progress (2016–April 2025)

1. Review Question

What are the key design strategies, translational outcomes, and emerging platforms in advanced drug delivery systems (DDS) reported in preclinical and clinical studies between 2016 and 2025?

2. Eligibility Criteria (PICOS)

Element	Inclusion Criteria	Exclusion Criteria
Population	Not applicable (DDS-focused review). Studies must involve a defined DDS platform (nanoparticles, micelles, implants, etc.).	Studies without a clear DDS component (e.g., pure drug formulations, medical devices without nanocarriers).
Intervention	Any advanced DDS: lipid nanoparticles, polymeric NPs, extracellular vesicles, microneedles, 3D-printed systems, etc.	Conventional formulations (solutions, tablets without engineered carriers).
Comparison	Optional (e.g., DDS vs. free drug, different DDS designs, routes of administration).	No comparison required for inclusion.
Outcomes	Characterization data, *in vitro*/*in vivo* efficacy, safety, pharmacokinetics, clinical endpoints, translational relevance.	Studies lacking key DDS characterization (size, charge, composition) or without biological/clinical outcome data.
Study Design	Original research: experimental studies, preclinical (*in vivo*, *ex vivo*), clinical trials (Phases 1–4).	Reviews, editorials, opinions, protocols, patents, *in silico*-only studies without experimental validation, non-English publications.
Time Frame	January 2016 – April 2025.	Published before 2016.

3. Information Sources & Search Strategy

Databases: PubMed/MEDLINE, Scopus, Web of Science.

Search Date: Literature search was conducted on 15 April 2025.

Search Terms: Combinations of:

Nanocarriers: "lipid nanoparticle", "liposome", "polymeric nanoparticle", "micelle", "exosome", "extracellular vesicle".

Platforms: "microneedle", "3D printed drug delivery", "implantable DDS".

Routes: "intranasal delivery", "transdermal", "oral delivery", "pulmonary delivery".

Cargo: "mRNA delivery", "siRNA", "CRISPR", "protein delivery".

Filters: English language, 2016–2025, original articles.

Supplementary: Hand-searching of reference lists of relevant reviews.

4. Study Selection Process

Duplicate Removal: using Reference Manager (e.g., EndNote, Zotero).

Title/Abstract Screening: two independent reviewers apply inclusion/exclusion criteria (A.U. & E.Y.).

Full-Text Assessment: remaining articles evaluated in detail against PICOS criteria.
Disagreements: Resolved by discussion or third reviewer adjudication (Zh.M.).
Flow Diagram: PRISMA 2020 flowchart will document the selection process.
5. Data Extraction
Standardized form in Excel/Google Sheets will include:
Study ID: Author, year, journal.
DDS Platform: Type, composition, size, charge.
Therapeutic Cargo: Drug, nucleic acid, protein, etc.
Route of Administration: IV, oral, intranasal, etc.
Study Model: *In vitro*, animal model, clinical trial phase.
Key Outcomes: Efficacy, pharmacokinetics, safety.
Translational Relevance: Clinical implications, scalability, regulatory aspects.

6. Quality Assessment
Preclinical Studies: Adapted from SYRCLE's risk of bias tool.
Clinical Trials: Cochrane Risk of Bias tool (RoB 2).
Focus: Study design, reporting of DDS characterization, statistical methods, reproducibility.

7. Data Synthesis
Narrative Synthesis: Thematic analysis of DDS platforms, mechanisms, routes, and outcomes.
Tabular Summary: Summary table of included studies (Supplementary Material S3).
Visualization: PRISMA flowchart, tables, conceptual diagrams.
