# Supplementary material for: A Systematic Review of Advanced Drug Delivery Systems: Engineering Strategies, Barrier Penetration, and Clinical Progress (2016–April 2025)
Source: Pharmaceutics. 2025 Dec 22;18(1):11. doi: 10.3390/pharmaceutics18010011 (PMC12845006; doi:10.3390/pharmaceutics18010011)
Supplement: Supplementary file 1 [file pharmaceutics-18-00011-s001.zip › Supplementary File S2 Full Electronic Search Strategy.pdf]

Supplementary File S2: Full Electronic Search Strategy

For the Systematic Review: " A systematic review of advanced drug delivery systems: engineering strategies, barrier penetration, and clinical progress (2016–April 2025)"

Date of Last Search: 15 April 2025

Databases Searched: PubMed/MEDLINE, Scopus, Web of Science Core Collection

Time Frame: 1 January 2016 – 15 April 2025

Language Restriction: English

Filters: Original research articles only; reviews, editorials, patents, conference papers excluded via syntax.

1. PubMed/MEDLINE (via NCBI)

Search Date: 15 April 2025

Records Retrieved: 1,842

```
(
  (
    "Drug Delivery Systems"[MeSH] OR
    "Nanoparticles"[MeSH] OR
    "Liposomes"[MeSH] OR
    "Exosomes"[MeSH] OR
    "Microfluidics"[MeSH] OR
    "Administration, Intranasal"[MeSH] OR
    "Microneedles"[MeSH] OR
    "Printing, Three-Dimensional"[MeSH]
  )
  OR
  (
    nanocarrier*[tiab] OR "nanoparticle*"[tiab] OR "lipid nanoparticle*"[tiab] OR "LNP"[tiab] OR "polymeric nanoparticle*"[tiab] OR
    liposome*[tiab] OR exosome*[tiab] OR "extracellular vesicle*"[tiab] OR biomimetic[tiab] OR
    "drug delivery"[tiab] OR "targeted delivery"[tiab] OR "controlled release"[tiab] OR "sustained release"[tiab] OR
    microneedle*[tiab] OR "3D print*"[tiab] OR implantable[tiab] OR microfluidic*[tiab] OR
```

```
   "intranasal delivery"[tiab] OR "nose-to-brain"[tiab] OR "pulmonary delivery"[tiab] OR transdermal[tiab] OR
   "stealth effect"[tiab] OR "protein corona"[tiab] OR "mucus penetration"[tiab]
  )
)
AND ("2016/01/01"[Date - Publication] : "2025/02"[Date - Publication])
AND english[la]
NOT (review[ptyp] OR editorial[ptyp] OR comment[ptyp] OR patent[ptyp])
```

2. Scopus (Elsevier)

Search Date: 15 April 2025

Records Retrieved: 1,933

```
TITLE-ABS-KEY
(
  (
   nanocarrier* OR "lipid nanoparticle*" OR "polymeric nanoparticle*" OR liposome* OR exosome* OR
   "extracellular vesicle*" OR biomimetic* OR microneedle* OR "3D print*" OR "implantable drug delivery" OR
   "microfluidic production" OR "intranasal delivery" OR "nose-to-brain" OR "pulmonary delivery" OR
   "transdermal delivery" OR "stealth effect" OR "protein corona" OR "mucus penetration"
  )
  AND
  (
   therapeutic* OR drug OR mrna OR sirna OR crispr OR "gene therapy" OR vaccine OR
   "cancer therapy" OR "clinical trial" OR "in vivo" OR translational
  )
)
AND PUBYEAR = 2016 OR PUBYEAR = 2017 OR PUBYEAR = 2018 OR PUBYEAR = 2019 OR PUBYEAR = 2020 OR
   PUBYEAR = 2021 OR PUBYEAR = 2022 OR PUBYEAR = 2023 OR PUBYEAR = 2024 OR PUBYEAR = 2025
AND (LIMIT-TO(LANGUAGE,"English"))
AND (LIMIT-TO(DOCTYPE,"ar"))  /* Articles only */
```

AND NOT (DOCTYPE("re") OR DOCTYPE("ed") OR DOCTYPE("cp")) /* Exclude reviews, editorials, conference papers */

3. Web of Science Core Collection (Clarivate)

Search Date: 15 April 2025

Records Retrieved: 1,075

#1: TS=(nanocarrier* OR "lipid nanoparticle*" OR "polymeric nanoparticle*" OR liposome* OR exosome* OR "extracellular vesicle*" OR biomimetic* OR microneedle* OR "3D print*" OR "implantable drug delivery" OR "microfluidic production" OR "intranasal delivery" OR "nose-to-brain" OR "pulmonary delivery" OR "transdermal delivery" OR "stealth effect" OR "protein corona" OR "mucus penetration")

#2: TS=(therapeutic* OR drug OR mrna OR sirna OR crispr OR "gene therapy" OR vaccine OR "cancer therapy" OR "clinical trial" OR "in vivo" OR translational)

#3: #1 AND #2

#4: PY=(2016-2025)

#5: LA=(English)

#6: DT=(Article)

#7: #3 AND #4 AND #5 AND #6

NOT DT=("Review" OR "Editorial Material" OR "Patent" OR "Meeting Abstract")

#8: #7

Total records identified: 1,842 (PubMed) + 1,933 (Scopus) + 1,075 (WoS) = 4,850
