# Supplementary material for: A Systematic Review of Advanced Drug Delivery Systems: Engineering Strategies, Barrier Penetration, and Clinical Progress (2016–April 2025)
Source: Pharmaceutics. 2025 Dec 22;18(1):11. doi: 10.3390/pharmaceutics18010011 (PMC12845006; doi:10.3390/pharmaceutics18010011)
Supplement: Supplementary file 1 [file pharmaceutics-18-00011-s001.zip › Supplementary File S4 Promising drug delivery platforms in advanced clinical development.pdf]

Supplementary File S4: Promising Drug Delivery Platforms in Advanced Clinical Development

Category	Platform / Candidate	Stage / Model	Indication(s)	Key Finding (Headline Benefit)	Significance / Reference
**A. HUMAN CLINICAL TRIALS**	Influenza Vaccine Microneedle Patch (TIV-MNP)	Phase 1 Trial	Seasonal Influenza Prevention	Safe, immunogenic (comparable to intramuscular injection), and highly acceptable. Minimal pain, potential for self-administration.	First-in-human clinical validation of a dissolving microneedle patch for vaccination. Paves the way for a pain-free, logistically simplified vaccine delivery. **Rouphael, N.G. et al., 2017** [30]
**B. PRECLINICAL DEVELOPMENT (CNS Targeting)**	Bilosomal Mucoadhesive In situ Gel (Zolmitriptan)	In vivo PK (rodents)	Migraine (Brain Targeting)	Increased brain bioavailability by >10-fold vs. IV injection. ~98% direct nose-to-brain transport. Significantly prolonged nasal residence time.	Demonstrates a potent integrated formulation strategy (vesicles + gel) to overcome nasal clearance and achieve highly efficient direct CNS delivery for small molecules. **El Taweel, M.M. et al., 2021** [31]
	RVG29-Targeted PLGA Nanoparticles (Intranasal)	In vivo biodistribution (rodents)	CNS Diseases (Proof-of-Concept)	Enhanced delivery to the trigeminal nerve and adjacent brain regions (striatum, brainstem) after intranasal administration, demonstrating spatial targeting.	Proof-of-concept for ligand-mediated targeting to spatially guide intranasal delivery within the CNS. **Chung, E.P. et al., 2020** [45]
	Multifunctional Chitosan Micelles (VGF plasmid)	In vivo (mice)	CNS Gene Delivery	Intranasal delivery achieved significantly higher brain transgene expression vs. intravenous route. Ligand-tagging enhanced targeting.	Demonstrates potential of engineered polymeric micelles for effective nose-to-brain gene delivery. **Lamptey, R.N.L. et al., 2022** [46]
	Ascorbic Acid-Conjugated PCL Nanoparticles (Paclitaxel)	In vivo PK (rats)	Brain Tumor Targeting	Intranasal administration resulted in a 5.6-fold increase in brain	Showcases a ligand-targeted, intranasal polymeric nanosystem for enhanced brain delivery of

				paclitaxel concentration compared to control.	chemotherapeutics. **Deshmukh, V. et al., 2025** [47]
**B. PRECLINICAL DEVELOPMENT (Oral Delivery of Biologics) B. PRECLINICAL DEVELOPMENT (Oral Delivery of Biologics)**	Albumin-PEG NPs with Permeation Enhancer (Bevacizumab)	In vivo (rats)	Oral Delivery of mAbs	Oral bioavailability reached 3.7% (1000-fold increase vs. control). Combinatorial strategy enabled significant intestinal absorption.	Breakthrough proof-of-concept for enabling oral delivery of monoclonal antibodies via rational nanoparticle design. **Pangua, C. et al., 2023** [32]
	Anionic Silica Nanoparticles (Oral)	In vivo (mice)	Oral Delivery of Peptides/Proteins	Enabled oral delivery of insulin with ~30% bioactivity vs. subcutaneous injection via reversible, integrin-mediated tight junction opening.	Novel, mechanism-driven approach using simple NPs to overcome the intestinal barrier for biologics. **Lamson, N. et al., 2019** [52]
**B. PRECLINICAL DEVELOPMENT (Ocular Delivery)**	Bilayer Dissolving Microneedles with PLGA Nanoparticles (Ocular)	Ex vivo / In vitro	Posterior Segment Eye Diseases (nAMD, DME)	Enabled sustained protein release for >2 months and effective transscleral localization of nanoparticles, bypassing invasive intravitreal injections.	Minimally invasive, sustained-release alternative to frequent intravitreal injections for chronic retinal diseases. **Wu, Y. et al., 2021** [37]
**B. PRECLINICAL DEVELOPMENT (Cancer Immunotherapy)**	Prodrug-based Nano-suspension (R848/HA-Toco)	Preclinical (Large Animal)	Immunotherapy (Head & Neck Cancer, Mast Cell Tumors)	Subcutaneous delivery formed a local depot, minimizing systemic toxicity of a potent TLR7/8 agonist while inducing anti-tumor immunity (67% response rate in canine tumors).	Addresses critical safety barrier by localizing immune stimulation. Strong translational data from a spontaneous large animal tumor model. **Lu, R. et al., 2019** [67]
**B. PRECLINICAL DEVELOPMENT (Advanced & Biomimetic Platforms)**	"Cellnex" Cell Surface Engineering	In vivo proof-of-concept	Enhanced Cell Therapy & Targeted Delivery	Coating living cells with nanocomplexes enhanced in vivo targeting (11-fold lung delivery) and	Paradigm shift: transforms cells into programmable, enhanced delivery vehicles, bridging nanomedicine and

				therapeutic function (improved checkpoint inhibitor efficacy).	advanced cell therapies. **Zhao, Z. et al., 2020** [33]
	Tunable Leukocyte-Membrane Coated NPs	In vivo (inflammation models)	Inflammatory Diseases	Increasing protein-to-lipid ratio in coatings directly enhanced nanoparticle targeting to inflamed endothelium in vivo, enabling tunable bioactivity.	Advances biomimetic design by establishing a quantitative, tunable parameter to rationally optimize targeting. **Zinger, A. et al., 2021** [34]
**C. ENABLING TECHNOLOGIES & TRANSLATIONAL SOLUTIONS**	Microfluidic Synthesis Platform for LNPs (Size-Controlled)	In vitro process optimization	RNA Therapeutics Manufacturing	Precise control enables tunable production of LNPs within narrow, optimized size range (60–120 nm), directly determining encapsulation efficiency and in vitro potency.	Provides critical, reproducible, and scalable manufacturing solution—a key translational bottleneck for GMP production. **Okuda, K. et al., 2022** [64]
	Predictive Stability Model for LNP-mRNA Vaccines	In silico / Translational Solution	Global Vaccine Logistics & Access	Kinetic model uses real-time temperature data to predict mRNA vaccine CQA loss and remaining shelf life, reducing waste.	Crucial digital tool to overcome the cold-chain barrier, enabling more efficient and equitable global distribution. **Kis, Z., 2022** [35]
